# Pressure-Derived Indices in the Left Main Coronary Artery: Insights From Comprehensive In Vivo Hemodynamic Studies of Diseased and Unobstructed Vessels

**DOI:** 10.1161/CIRCINTERVENTIONS.125.015320

**Published:** 2025-05-12

**Authors:** Ozan M. Demir, Aish Sinha, Haseeb Rahman, Matthew Ryan, Kevin O’Gallagher, Howard Ellis, Matthew Li Kam Wa, Smriti Saraf, Khaled Alfakih, Ian Webb, Narbeh Melikian, Kalpa De Silva, Amedeo Chiribiri, Sven Plein, Divaka Perera

**Affiliations:** 1 British Heart Foundation Center of Excellence and National Institute for Health Research Biomedical Research Center at the School of Cardiovascular Medicine and Sciences, King’s College London, United Kingdom (O.M.D., A.S., H.R., M.R., K.O.G., H.E., M.L.K.W., K.A., I.W., N.M., K.D.S., A.C., S.P., D.P.).; 2 Guy’s and St Thomas’ NHS Foundation Trust, London, United Kingdom (O.M.D., A.S., H.R., H.E., M.L.K.W., K.D.S., A.C., D.P.).; 3 King’s College Hospital NHS Foundation Trust, London, United Kingdom (M.R., K.O.G., K.A., I.W., N.M.).; 4 East Kent Hospitals NHS Foundation Trust, United Kingdom (S.S.).

**Keywords:** coronary artery disease, fractional flow reserve, heart, humans, plaque

## Abstract

**BACKGROUND:**

Pressure-based physiological evaluation of coronary artery disease is well established, but its application is limited in left main coronary artery (LMCA) disease. Our aim was to investigate whether pressure-based indices are comparable in the left anterior descending (LAD) and left circumflex artery (LCx) branches of the LMCA, and if discordance is due to differences in microvascular function in these territories.

**METHODS:**

Simultaneous measurements of coronary pressure and flow were made in patients with (1) isolated LMCA disease and (2) unobstructed coronary arteries. Fractional flow reserve, instantaneous wave-free ratio, and microvascular resistance reserve values in the LAD were compared with those of the LCx.

**RESULTS:**

A total of 80 patients were enrolled (mean age 65±10 years, 56% male). In those with isolated LMCA disease, fractional flow reserve in the LAD was lower than in the LCx (0.74±0.11 versus 0.81±0.11; *P*<0.0001). Instantaneous wave-free ratio was also lower in the LAD (0.89 [0.76–0.92] versus 0.94 [0.88–0.97]; *P*<0.0001). The misclassification rates of functionally significant coronary disease, when these indices were measured in the LCx, were 21% for fractional flow reserve and 28% for instantaneous wave-free ratio. Microvascular resistance reserve was higher in the LAD than the LCx, in cohorts with diseased (3.57±1.40 versus 2.50±0.81; *P*<0.0001) or unobstructed LMCA (3.40±0.78 versus 2.47±0.68; *P*<0.0001). Microvascular resistance reserve in the LAD territory was similar regardless of whether the LMCA was obstructed or not (*P*=0.56). Similarly, microvascular resistance reserve in the LCx territory was comparable between cohorts (*P*=0.88).

**CONCLUSIONS:**

Microvascular resistance in the LAD is lower than in the LCx territory. Consequently, fractional flow reserve and nonhyperemic pressure-derived indices are lower in the LAD than the LCx. These findings have important implications for how LMCA atheroma should be assessed in clinical practice and also suggest the need for territory-specific thresholds for defining abnormal microvascular function and epicardial conductance.

**WHAT IS KNOWN**

- Pressure-based physiological evaluation of coronary artery disease is well established, including in the presence of left main coronary artery disease.
- Current clinical practice generally assumes these pressure-based indices are vessel-agnostic, applying the same diagnostic thresholds to all coronary vessels.

**WHAT THE STUDY ADDS**

- Invasive physiological indices differ significantly between the left anterior descending artery and the left circumflex artery due to inherent differences in microvascular resistance and coronary flow.
- These findings were observed both in patients with isolated left main coronary artery disease and in patients without epicardial or microvascular disease, indicating that they are intrinsic to the coronary circulation.
- In the assessment of left main coronary artery disease, physiological evaluation should prioritize left anterior descending artery-based measurements, as relying solely on left circumflex artery measurements could result in significant left main coronary artery disease being missed in over one-fifth of cases. A comprehensive approach should include hyperemic pressure-wire pullback and intracoronary imaging in the presence of significant downstream disease.

Atheroma affecting the left main coronary artery (LMCA) is considered to be the most prognostically impactful of the entire spectrum of coronary disease.^1^ However, while intracoronary pressure-based metrics (like fractional flow reserve [FFR] or instantaneous wave-free ratio [iFR]) are considered the standard of care in determining the functional significance of coronary lesions and the requirement for revascularization, their use in LMCA disease is paradoxically low.^2–6^ This primarily relates to uncertainty about how to interrogate this important coronary bifurcation and which daughter branch (or branches) should be assessed during physiological testing. It is thought that atheroma in the LMCA affects flow in the daughter branches equally and hence, that FFR (or iFR) in both limbs of the LMCA would be similar. The assumed parity of pressure-based indices also implies that microvascular resistances in the left anterior descending artery (LAD) and left circumflex artery (LCx) are comparable. However, previous studies have suggested that indices of microvascular resistance and coronary flow are different between coronary territories.^7,8^ Therefore, it remains unclear whether pressure-based indices are also disparate between coronary territories.

**See Editorial by Ahn and Fearon**

Our primary hypothesis was that minimal and resting microvascular resistence in the LAD is different from that in the LCx; as a consequence, FFR (and iFR) would be different in the LAD and LCx in patients with isolated LMCA disease. Our second hypothesis was that differences in microvascular function between LAD and LCx are an innate property of the coronary circulation and would be found even in patients with unobstructed coronary arteries.

To address these hypotheses, we studied 2 cohorts of patients in whom we characterized microvascular function in the LAD and LCx territories, using the recently described index of microvascular resistance reserve (MRR),^9–12^ a measure that is independent of epicardial disease. The first cohort consisted of patients with disease isolated to the LMCA, and the second cohort consisted of patients with unobstructed epicardial arteries.

## Methods

The data that support the findings of this study are available from the corresponding author upon reasonable request.

### Study Population

Patients were prospectively enrolled at the 2 campuses of King’s College London (St Thomas’ Hospital NHS Foundation Trust and King’s College Hospital NHS Foundation Trust) if they had a clinical indication for diagnostic coronary angiography (such as chest pain or exertional dyspnea) or were scheduled for percutaneous coronary intervention.

Patients were eligible for inclusion in the Isolated LMCA Disease cohort, if they had atheroma that was angiographically and physiologically isolated to the LMCA, predefined by (1) FFR pullback gradient ≤0.05 FFR units in both LAD and LCx, and (2) where the LMCA lesion contributed to ≥80% of the total FFR in each vessel. Patients were eligible for inclusion in the Unobstructed LMCA cohort if they had: (1) angiographically normal vessels with no stenosis; (2) normal epicardial conductance (FFR>0.80 in both branches); and (3) normal microvascular function (LAD coronary flow reserve ≥2.0). Hence, the Unobstructed LMCA cohort constitutes a reference cohort with normal coronary circulation, both anatomically and physiologically. Exclusion criteria for both cohorts were: (1) left ventricular ejection fraction <50%; (2) recent acute coronary syndrome (within 4 weeks); (3) concomitant valvular heart disease (greater than mild on echocardiography); (4) resting heart rate >120 bpm; (5) previous coronary artery bypass surgery; and (6) contraindication or intolerance to adenosine or contrast. The study protocols were approved by the UK National Research Ethics Service (20/LO/0245 and 20/LO/1294). All participants provided written informed consent before enrollment.

### Cardiac Catheterization Protocol

Cardiac catheterization was conducted via the right radial artery using standard coronary catheters. All patients received 1 mg intravenous midazolam and intraarterial unfractionated heparin (70 U/kg) before intracoronary physiological measurements. Intracoronary isosorbide dinitrate was administered (400–600 µg into the left coronary system) before undertaking physiology measurements. The physiology protocol included measurement of distal coronary pressure ratios (Verrata Plus Volcano 0.014-inch intracoronary wire; Philips Healthcare, Amsterdam, NL) followed by manual steady-speed pressure-wire pullback along the length of each vessel. In a subset, coronary flow was simultaneously measured using a Doppler/pressure sensing guidewire (Combowire, Philips Volcano, CA), positioned in the same angiographic location as the previous pressure-wire recording. Measurements were made at steady state, at rest, and during hyperaemia induced using intravenous adenosine at a rate of 140 mcg/kg/min, in both main daughter branches of the LMCA. If there was pressure-wire drift >0.02 units, repeat measurements were performed.

### Analysis of Coronary Physiological Data

Coronary Doppler-based data were exported into a custom-made study manager program (Academic Medical Center, University of Amsterdam, the Netherlands) and analyzed on custom-made software, Cardiac Waves (King’s College London, United Kingdom).^13^ Doppler peak flow velocities were averaged over ≥3 consecutive heartbeats to derive average peak velocity. FFR was defined as the ratio of distal coronary to aortic pressure during maximal hyperemia.^14^ Pd/Pa was defined as ratio of resting distal coronary pressure to aortic pressure. Instantaneous wave-free ratio was defined as Pd/Pa during the latter 75% of diastole, excluding the terminal 5 ms.^15^ Coronary flow velocity reserve (CFvR) was defined as the ratio of hyperemic to resting average peak velocity.^16^ MRR was calculated as CFvR divided by FFR, multiplied by basal aortic pressure divided by hyperemic aortic pressure.^11^ Hyperemic microvascular resistance was calculated as distal coronary pressure divided by flow velocity during the maximal hyperemic condition.^17^

Sensitivity analyses were performed to assess: (1) the potential influence of hydrostatic forces on intracoronary pressure measurements (due to differences in the height of the distal sensor compared with the proximal sensor^18^) using computed tomography coronary angiograms (Supplemental Appendix S1)^19,20^; (2) the relationship between subtended myocardial mass and MRR measurements on a per-vessel basis, with mass estimated by cardiac magnetic resonance imaging (Achieva; Philips Healthcare, the Netherlands; Supplemental Appendix S2)^21,22^; and (3) pressure wire-derived indices by anatomic location of LMCA stenosis (Supplemental Appendix S3).

### Statistical Analyses

Our primary hypothesis was that, in patients with isolated LMCA stenosis, FFR in the LAD will be lower than in the LCx. Forty-four patients will provide 90% power (α=0.05) to detect a minimum difference in FFR between vessels of 0.05 FFR units (at a significance level of 5%, assuming a SD of 0.10 FFR units). A paired within-group comparison was performed for each pressure-derived index (FFR, iFR) using either Student *t* tests or the Wilcoxon matched test, depending on the normality of the data. Our second (hierarchical) hypothesis was that the MRR will be higher in the LAD compared with the LCx. Nineteen patients will provide 90% statistical power to detect a minimum difference in MRR between vessels of 0.8 MRR units (at a significance level of 5%, assuming a SD of 1.0 MRR units). Given the hierarchical hypothesis testing, the significance level was not adjusted. To assess whether potential differences or similarities in microvascular function were independent of epicardial disease and specific to the vascular bed, we performed between-group comparisons for each vessel (comparing per-vessel values in the LMCA disease cohort versus those in the unobstructed epicardial arteries cohort).

Misclassification of LMCA disease severity (in the Isolated LMCA Disease cohort) was calculated for hyperemic and nonhyperemic indices, using the LAD as the reference standard, and comparing within-cohort to the LCx measurements, using routinely used binary thresholds (FFR ≤0.80 and iFR ≤0.89).

The normality of the data was evaluated using the construction of a histogram, normal quantile-quantile (Q-Q) plot, and the Shapiro-Wilk test. Continuous data that followed a normal distribution were reported as mean±SD and analyzed using paired Student *t* tests. Non-normally distributed data were described using the median and interquartile range and compared using paired Wilcoxon matched tests. Categorical variables were presented as frequency and percentages and analyzed using the χ^2^ test. Statistical analyses were performed using SPSS Software, version 29.0 (IBM, Somers, New York) and Prism Software, version 10.2.3 (GraphPad Software, San Diego, CA).

## Results

### Study Flow and Participant Characteristics

**Figure 1.**
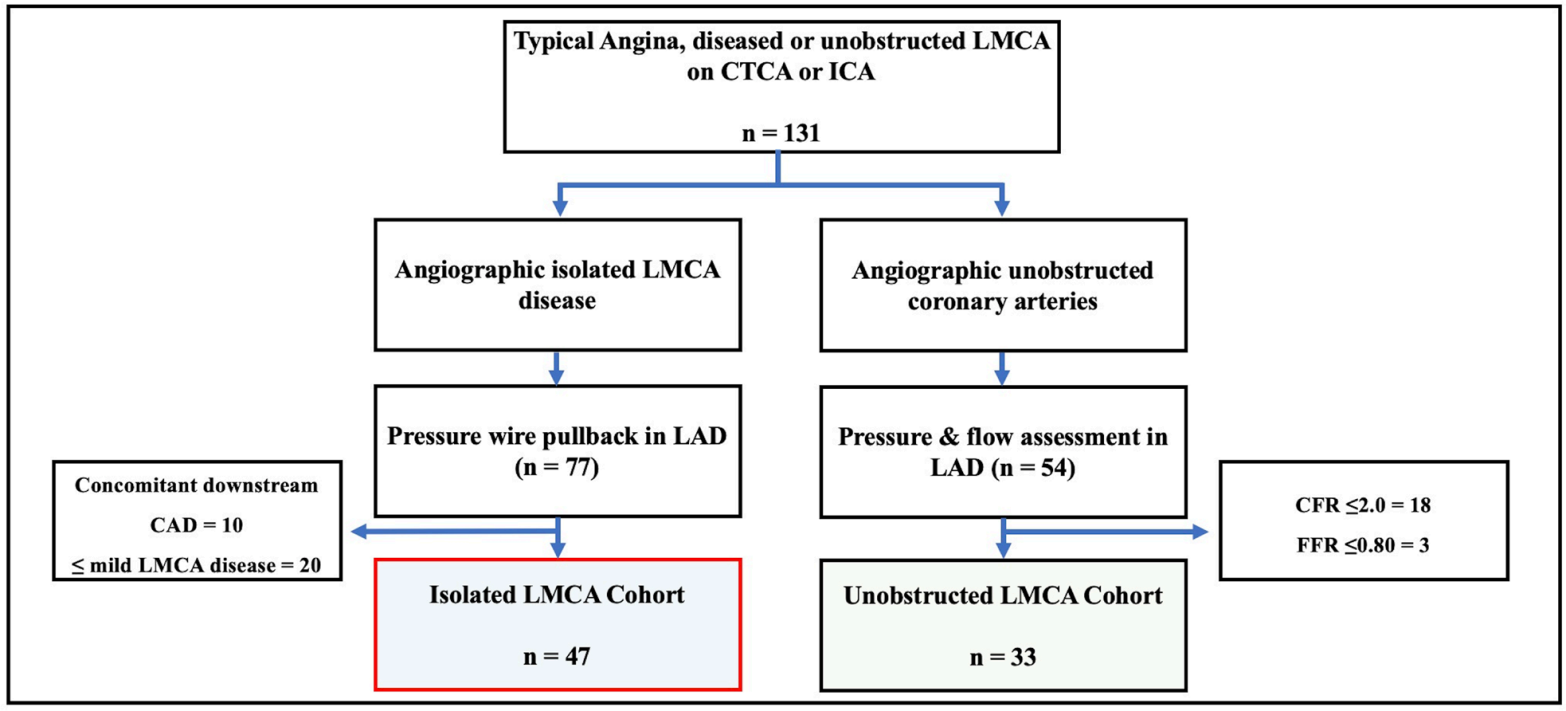
**Study outline.** CONSORT diagram. In the isolated left main coronary artery (LMCA) disease cohort, of the 47 patients included in the study, 22 patients underwent dual-pressure and Doppler assessment. All patients in the unobstructed LMCA cohort had dual-pressure and Doppler measurements. CAD indicates coronary artery disease; CFR, coronary flow reserve; CTCA, computed tomography coronary angiography; FFR, fractional flow reserve; ICA, invasive coronary angiogram; and LAD, left anterior descending artery.

Patients were prospectively recruited between July 30, 2019, and August 2, 2023. Seventy-seven patients were enrolled in the Isolated LMCA Disease cohort, but 20 were excluded due to ≤mild LMCA disease (including cases where a lesion was suspected on computed tomography coronary angiogram but not apparent on invasive angiography), and 10 excluded due to concomitant downstream coronary artery disease (Figure 1). Thus, a total of 47 patients were included in the Isolated LMCA Disease cohort, of which 22 patients had dual-pressure and Doppler assessment. Fifty-four patients were enrolled in the Unobstructed LMCA cohort, but 21 were excluded due to CFvR ≤2.0 or FFR ≤0.80. Thus, 33 patients were included in the Unobstructed LMCA cohort, all of whom underwent dual-pressure and Doppler assessment. Paired LAD and LCx coronary physiological measurements were performed in all patients. Table 1 presents the demographic and baseline clinical characteristics of both cohorts.

**Table 1.**
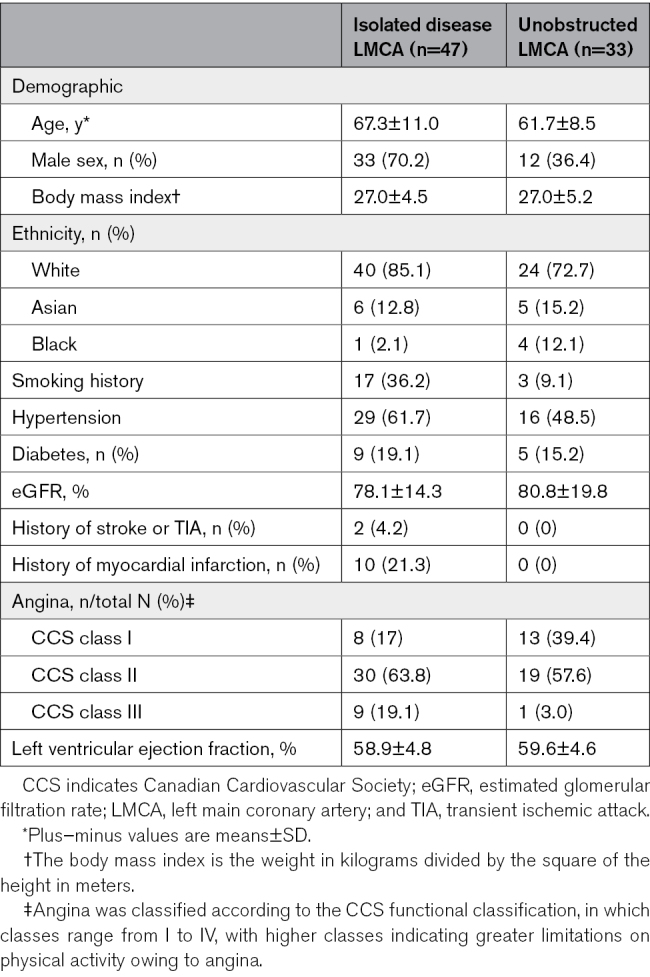
Demographic and Baseline Clinical Characteristics of Diseased and Unobstructed LMCA

### Pressure-Derived Indices in the LAD Versus LCx

In patients with isolated LMCA disease, the LMCA on quantitative coronary angiography was 4.52±0.58 mm, and the diameter stenosis was 52±15%. FFR was 0.74±0.11 in the LAD and 0.81±0.11 in the LCx (*P*<0.0001; Table 2). iFR values were also lower in the LAD than in the LCx (0.89 [0.76–0.92] versus 0.94 [0.88–0.97]; *P*<0.0001). A similar difference was seen with whole-cycle Pd/Pa ratio (Table 2). Furthermore, pressure-wire pullback evaluation demonstrated that the pressure drop was higher between the LMCA ostium and the LAD ostium compared with the pressure drop between the LMCA ostium and the LCx ostium; the pressure gradient between the distal vessel and ostium was minimal and similar between the LAD and LCx, confirming the absence of functionally significant downstream disease.

**Table 2.**
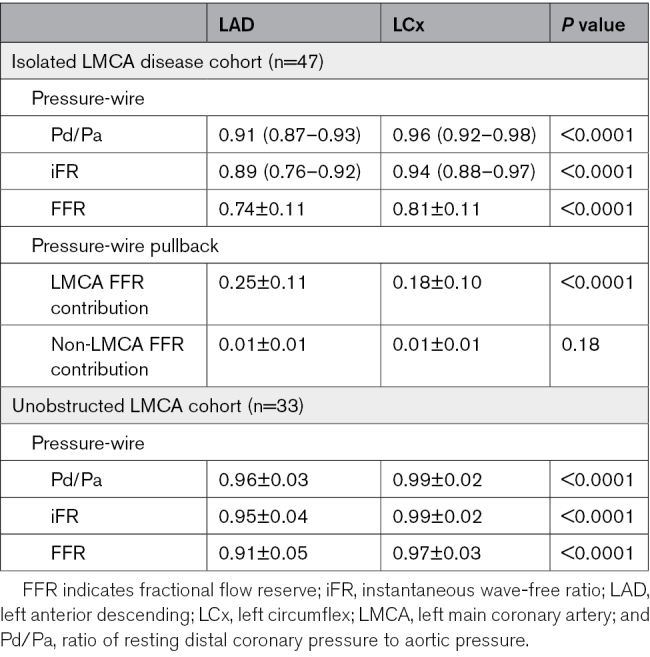
Pressure-Derived Physiological Assessment

**Figure 2.**
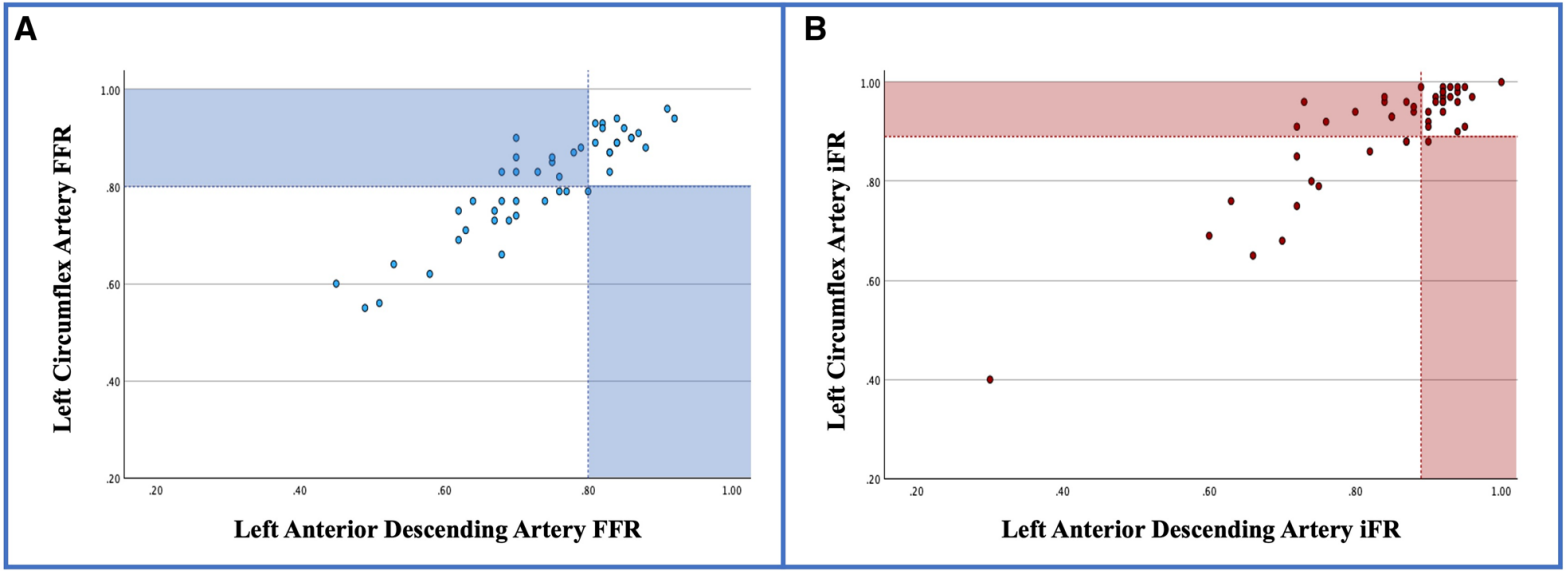
**Correlation of pressure wire-derived indices in the left anterior descending and left circumflex arteries.** Within-cohort coefficient of determination (*R*^2^) analyses of (**A**) fractional flow reserve (FFR) and (**B**) instantaneous wave-free ratio (iFR) demonstrated FFR *r*^2^=0.83 and iFR *r*^2^=0.81 (*P*<0.0001 for both). The dashed lines represent the respective cutoff values for FFR (≤0.80) and iFR (≤0.89). Shaded quadrants represent misclassification.

The rate of misclassification of functional significance in the isolated LMCA disease patients, if based on measurements performed in the LCx, was 21% when using FFR and 28% for iFR. All instances of misclassification were due to a significant LMCA lesion being incorrectly judged as not significant (Figure 2).

Of the 33 patients in the unobstructed LMCA cohort, 15 had computed tomography coronary angiography performed. When these measurements were used to adjust for the impact of hydrostatic forces on FFR values, the FFR values in the LAD remained lower than in the LCx (Supplemental Appendix S1). Furthermore, computed tomography confirmed the absence of atheromatous disease, substantiating invasive anatomic and physiological findings.

### Microvascular Function in LAD and LCx

**Figure 3.**
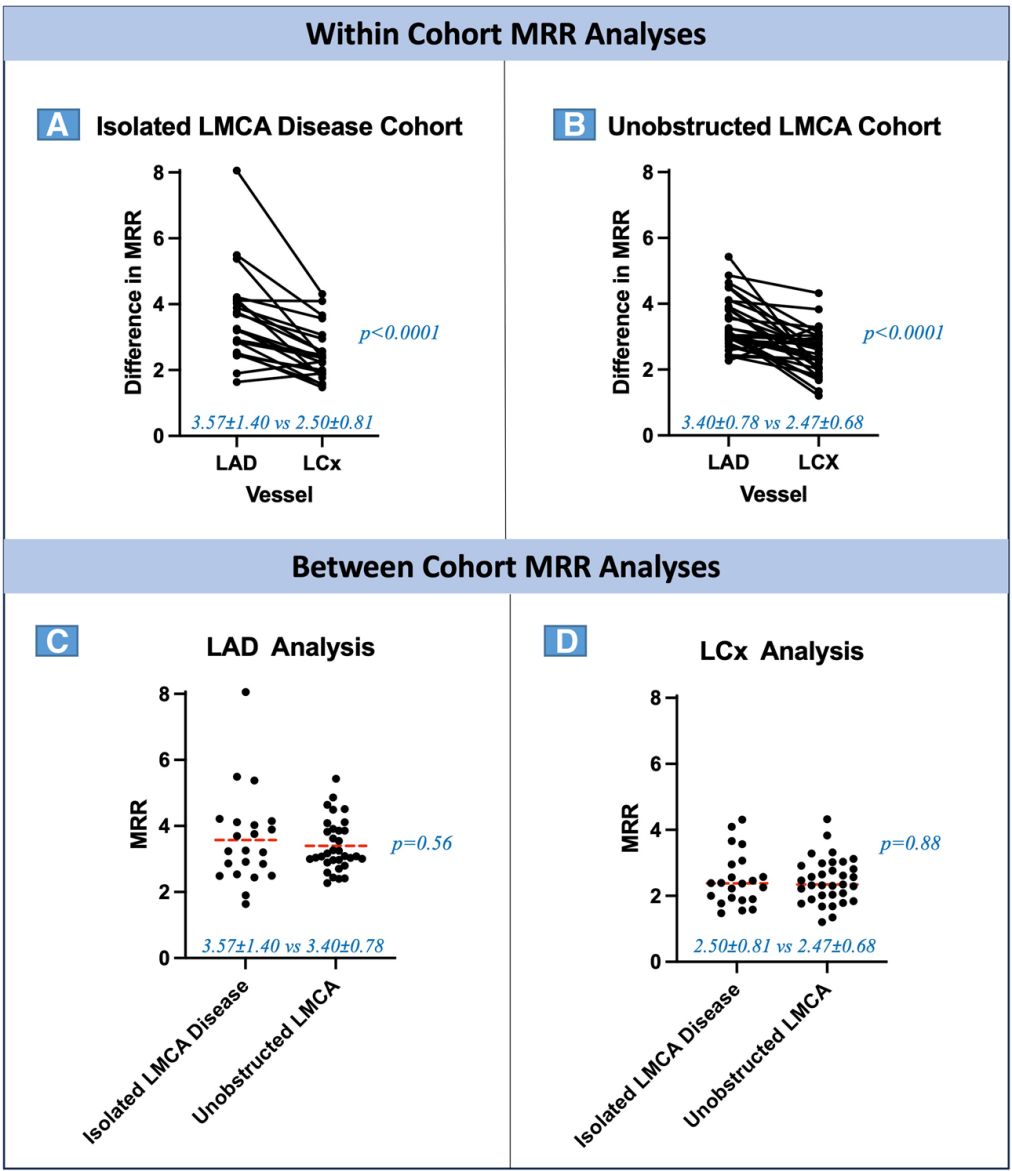
**Within- and between-cohort microvascular resistance reserve (MRR) analyses.** Within-cohort analyses are shown in **A** and **B**. Between-cohort analyses are shown in **C** and **D**. The dashed red lines depict mean values. LAD indicates left anterior descending; LCx, left circumflex artery; and LMCA, left main coronary artery.

In the within-cohort analysis, MRR values were higher in the LAD than in the LCx, both in patients with a diseased LMCA (3.57±1.40 versus 2.50±0.81; *P*<0.0001) and in those with unobstructed LMCA (3.40±0.78 versus 2.47±0.68; *P*<0.0001). Similarly, minimal hyperemic microvascular resistance was lower in the LAD than in the LCx in patients with LMCA disease (1.93±0.49 versus 2.81±0.66 mm Hg·cm^−^1·s^−^1; *P*<0.001) and in those with unobstructed LMCA (1.98±0.44 versus 2.93±1.30 mm Hg·cm^−^1·s^−^1; *P*<0.0001; Figure 3). Maximal coronary flow (hyperemic average peak velocity) and CFvR were higher in the LAD compared with the LCx, in both diseased and unobstructed LMCA cohorts (Table 3).

**Table 3.**
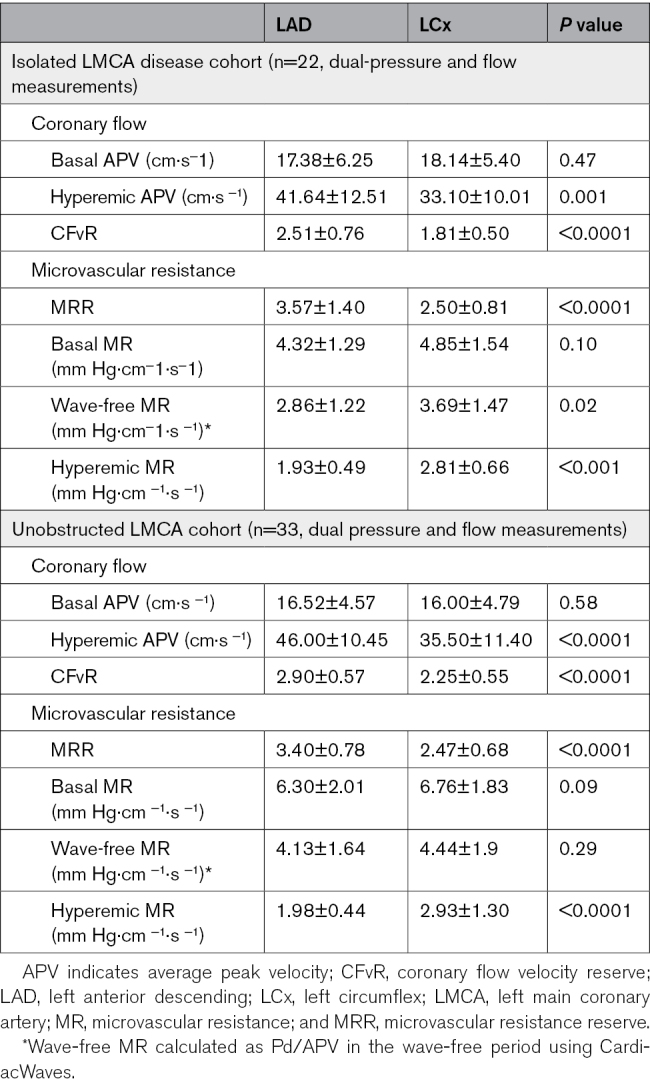
Flow-Based Indices in LAD Versus LCx Arteries

In the between-cohort analysis, MRR in the LAD territory was similar in patients with diseased or unobstructed LMCA (3.57±1.40 versus 3.40±0.78; *P*=0.56). Similarly, in the LCx territory, MRR was similar in patients with diseased LMCA compared with those with an unobstructed LMCA (2.50±0.81 versus 2.47±0.68; *P*=0.88). Minimal hyperemic microvascular resistance values were also similar for each vascular territory regardless of whether the patient had a diseased or unobstructed LMCA (LAD 1.93±0.49 versus 1.98±0.44 mm Hg·cm^−^1·s^−^1; *P*=0.71; LCx, 2.81±0.66 versus 2.93±1.30 mm Hg·cm^−^1·s^−^1; *P*=0.70).

Myocardial mass and MRR were correlated, with a coefficient of determination (*r*^2^) of 0.30 (*P*<0.0001; Supplemental Appendix S2).

## Discussion

The results of our study demonstrate, for the first time, that pressure-derived indices of epicardial disease are different in the LAD and LCx of patients with atheroma isolated to the LMCA. This is due to greater MRR and lower minimal microvascular resistance in the LAD territory than in the LCx. The clinical implication is that revascularization should be guided by physiological assessment of LMCA atheroma in the LAD artery, as relying solely on the LCx would risk functionally significant LMCA disease being missed in at least 1 in 5 cases. The other novel finding from our study is that the differences in microvascular function between LAD and LCx territories are an innate physiological property of the coronary circulation, with the same pattern of difference observed in patients with unobstructed coronary arteries as in those with isolated LMCA disease.

### Pressure-Derived Indices

We have demonstrated that routinely used invasive measures of epicardial coronary disease are different in each daughter branch of the LMCA. The pressure drop across a segment of vessel relates to flow velocity via a quadratic relationship, with flow velocity in turn being inversely related to microvascular resistance.^2^ Measuring microvascular resistance under different conditions and different phases of the cardiac cycle revealed the reason for the discrepant pressure drops in the 2 vessels: compared with the LCx, microvascular resistance in the LAD was lower at hyperemia (leading to a lower FFR), at rest during the wave-free period (leading to a lower iFR), and at rest when averaged out over the whole cardiac cycle (leading to a lower resting Pd/Pa). These findings challenge a key assumption that underlies the application of coronary physiological indices in contemporary practice, that the diagnostic threshold for each index is agnostic to the vessel being interrogated. This, in turn, has 2 major implications for pressure-derived indices. First, when evaluating the functional significance of LMCA atheroma, the classification of disease as hemodynamically significant or not (and subsequent management with bypass surgery, PCI, or medical therapy alone) may depend on which daughter branch is interrogated. In our study, the magnitude of discordance between the LAD and LCx for all commonly used pressure-derived indices (including FFR, iFR, and Pd/Pa) was large enough to fundamentally change revascularization decisions in a significant proportion of patients with left main atheroma; the LMCA disease appeared nonsignificant by contemporary binary thresholds when the LCx was assessed, despite being unequivocally significant when measurements were performed in the LAD in these cases. Second, the insights we have obtained from studying the diseased LMCA may apply even when assessing non-LMCA vessels by using coronary pressure ratios, both to identify hemodynamically significant disease and when these indices are used to evaluate the extent and quality of revascularization. This may explain the recently reported findings of a difference in post-PCI values of FFR in the LAD compared with the LCx (or right coronary artery) despite clinically similar outcomes in the medium term, indicating that the values of pressure-based indices that denote significant disease (or the adequacy of revascularization) may be specific to the vessel being interrogated.^23^

### Coronary Flow

Doppler transducers on intracoronary wires measure coronary flow velocity (in cm/s), which in turn is commonly used as surrogate of volumetric coronary flow (in cm^3^), although the latter also depends upon the cross-sectional area of the vessel at the point of the Doppler transducer. When comparing flow in a given artery under different conditions, this assumption is reasonable as the cross-sectional area of the epicardial vessel could be expected to change minimally between conditions; this is the case with coronary flow reserve measured using pharmacological agents (such as adenosine or papaverine), which have a dominant effect on the microcirculation rather than epicardial vessel caliber. However, when comparing flow in arteries of different sizes, the ratio of flow velocity may not correlate directly with the ratio of volumetric flow. Furthermore, flow-mediated dilation (and hence cross-sectional vessel area) may be different in epicardial arteries of different sizes. Therefore, despite our observation that flow velocity was comparable between the LAD and LCx, it is likely that volumetric flow is different between these vessels, which would also reflect the differences seen in resistances of the 2 beds.^24–26^ To this extent, our finding in humans mirrors the discordance between flow velocity and volumetric flow that was demonstrated in seminal animal studies^27,28^ and provides another mechanistic explanation for the difference in trans-lesional pressure drop between the LMCA and LAD versus LCx.^24–28^

### Microvascular Function

The capacity to decrease microvascular resistance in response to demand, as measured by MRR, was similar in a given vessel between patients with and without epicardial atheroma. This corroborates the fact that this index is minimally influenced by epicardial resistance and can thus be considered a specific index for the assessment of microvascular function. Akin to MRR, minimal microvascular resistance (quantified by hyperemic microvascular resistance in our study) was significantly lower in the LAD compared with the LCx (resulting in a higher CFvR in the LAD compared with the LCx) in both patient cohorts. This also has implications for multivessel assessment of microvascular function, as has been advocated in patients with angina but unobstructed coronary arteries.^29^ As such, the binary MRR and CFvR thresholds for diagnosing abnormal microvascular function may vary in the LAD, LCx, and right coronary artery. There are 2 potential mechanistic explanations for differential microvascular function across vascular beds. The first is that this relates to differences in the mass of subtended myocardium. Our findings lend some support to this, in that 30% of the variation in MRR (as denoted by a coefficient of variation of 0.30) seemed explicable by differences in subtended mass. The second possibility is that the LAD myocardium (per gram of tissue) may need to accommodate a greater dynamic range of oxygen requirements and hence blood flow demand, perhaps reflecting functional differences in the septum/apex versus the lateral wall of the left ventricle. We have no means of confirming or refuting the latter directly in our study, although it is notable that other studies modulating contractility strength and delay in these areas show variations in flow reserve and cardiac coronary coupling.^30^

Practically, how should the findings of this study affect the clinical application of pressure-derived indices of epicardial coronary disease? There is currently no consensus on how discordance between FFR (or iFR) values between the LAD and LCx should be resolved. A common contemporary interpretation is to regard a positive index in the LAD coupled with a negative value in the LCx as proof that the LMCA disease itself is nonsignificant, and hence that revascularization should focus on the LAD (especially if there is angiographically apparent atheroma in the LAD). Our findings provide a note of caution with this approach.

Based on our findings, we recommend 2 key approaches for the physiological assessment of the LMCA. First, pressure-wire assessment should be based on measurements in the LAD, as relying solely on LCx measurements could result in significant LMCA disease being missed in over one-fifth of cases. Second, downstream disease in the LAD should be excluded by performing a pressure-wire pullback—ideally under hyperemic conditions to optimize spatial resolution—rather than relying on concordance or discordance of FFR (or iFR) between the LAD and LCx. In the presence of functionally significant downstream disease, adjunctive intracoronary imaging could also be considered, rather than relying solely on physiological assessment of the LMCA-LCx (or other side branch) axis to rule out functional LMCA disease. However, in cases where the LAD is unusually small and the LCx is dominant, with a larger myocardial mass at risk, the opposite may be true. Finally, these findings may be extrapolated to non-LMCA pressure-wire-based assessments, in the presence of a bifurcation or side branch anatomy, advocating wire position in the target vessel. For example, in assessing proximal LAD disease pressure-wire placement in a diagonal branch may result in inaccurate LAD physiological adjudication.

### Study Limitations

This was a relatively small study designed to address specific mechanistic hypotheses and we do not yet have clinical outcome data relating to the application of our findings. Coronary luminal imaging was not performed to definitively exclude the presence of occult coronary artery disease. Consequently, in the Isolated LMCA Disease cohort, subtle extensions of LMCA disease into the LAD or LCx vessels may not have been fully identified, despite a comprehensive pullback pressure gradient assessment. However, sensitivity analysis within this cohort demonstrated consistent results across varying locations of isolated LMCA disease (ostial, midshaft, and distal LMCA).

### Conclusions

In patients with isolated LMCA disease, pressure wire-derived indices are lower in the LAD than in the LCx, driven by a higher flow rate and lower minimal microvascular resistance. Notably, these findings were also observed in patients without epicardial or microvascular coronary artery disease, suggesting that these differences are intrinsic to the hemodynamics of coronary circulation rather than a consequence of LMCA disease. This has important clinical implications for assessing the significance of LMCA disease and broader ramifications for the future application of these physiological indices, even in non-LMCA disease.

## Article Information

### Sources of Funding

This study was supported by the British Heart Foundation (PG/19/9/34228, RE/18/2/34213) and the UK National Institute for Health Research (through the Biomedical Research Center award to King’s College London and Guy’s and St Thomas’ Hospital).

### Disclosures

None.

### Supplemental Material

Supplemental Methods

Tables S1–S2

Figure S1

## Supplementary Material
