# Supplementary material for: Pressure-Derived Indices in the Left Main Coronary Artery: Insights From Comprehensive In Vivo Hemodynamic Studies of Diseased and Unobstructed Vessels
Source: Circ Cardiovasc Interv. 2025 May 12;18(7):e015320. doi: 10.1161/CIRCINTERVENTIONS.125.015320 (PMC12244971; doi:10.1161/CIRCINTERVENTIONS.125.015320)
Supplement: Supplementary file 1 [file hcv-18-e015320-s001.pdf]

SUPPLEMENTAL MATERIAL

Pressure derived indices in the left main coronary artery: insights from comprehensive in vivo hemodynamic studies of diseased and unobstructed vessels

S1. Computed tomography measurements and corrected FFR measurements in Unobstructed LMCA cohort

The potential influence of hydrostatic forces on intracoronary pressure measurements (due to differences in height of distal sensor compared to the proximal sensor) was evaluated using computed tomography coronary angiograms. All computed tomography coronary angiogram studies were performed using a third-generation dual-source CT (Siemens Healthcare, Forchheim, Germany) with ECG synchronization. The images were acquired according to the Society of Cardiovascular Computed Tomography guidelines. Coronary heights were calculated using an electronic radiology reporting program (OsiriX™ v13.0.2) as previously described. Corrected FFR measurements were calculated by multiplying the height (between the left coronary ostium and the measurement point in the LAD and LCx arteries) by 0.8, in accordance with Pascal's Law and adjusted for blood density. This calculation provided a positive or negative change in pressure (mmHg), which was subsequently added to the distal coronary pressure (Pd) measurement in the corresponding vessel.

Impact of hydrostatic forces was assessed, paired analysis of corrected FFR measurements, according to computed tomography coronary angiography height, was conducted.

Fifteen Unobstructed LMCA cohort patients had computed tomography coronary angiography performed and were included in the analysis. When these measurements were used to adjust for the impact of hydrostatic forces on FFR values, the FFR values in the LAD remained lower than the LCx. Furthermore, in these Unobstructed LMCA cohort patients, computed tomography confirmed the absence of atheromatous disease, substantiating invasive anatomical and physiological findings.

**Supplementary Table 1. Computed Tomography Measurements and FFR Measurements Corrected for Hydrostatic Pressure in LMCA Physiology Study**			
	LAD	LCx	P-value
**Height from LCA ostium on CT (mm)**			
Ostium	-0.2	-2.9	<0.001
Mid-vessel	10.2	-10	<0.001
Distal Vessel	25.6	-31	<0.001
**Correction Factor**			
FFR Correction Factor	+0.03 ± 0.01	-0.03 ± 0.01	<0.001
**Fractional Flow Reserve**			
Uncorrected	0.90 ± 0.04	0.99 ± 0.03	<0.001
Corrected	0.93 ± 0.04	0.97 ± 0.03	0.03

**LMCA denotes left main coronary artery; LAD, left anterior descending; LCx, left circumflex; FFR, fractional flow reserve; LCA, left coronary artery.*

S2. Per-vessel correlation between subtended myocardial mass and MRR measurements

Subtended myocardial mass per vessel was assessed using cardiac magnetic resonance (CMR). All high-resolution CMR scans were performed on a dedicated 3-Tesla CMR scanner (Achieva, Philips Healthcare, Netherlands). Contiguous short-axis slices were acquired from the base to the apex to calculate left ventricular function and mass (CVI42, v5.1.1, Circle Cardiovascular Imaging, Calgary, Ontario, Canada). Ventricular volumes were manually calculated from endocardial borders. The left ventricular epicardial border was also delineated to determine wall thickness, wall thickening and myocardial mass. The longitudinal extent of the LV cavity was delineated on a 4-chamber long-axis image, and the anterior and inferior right ventricular insertion points identified: CVI42 software subsequently output results for each myocardial segment according to the standard AHA model. The analysis was performed as per the Society for Cardiovascular Magnetic Resonance Standardized Image Interpretation guidelines.

The relationship between subtended myocardial mass and microvascular resistance reserve (MRR), on a per-vessel basis, was evaluated using coefficient of determination (r^2).

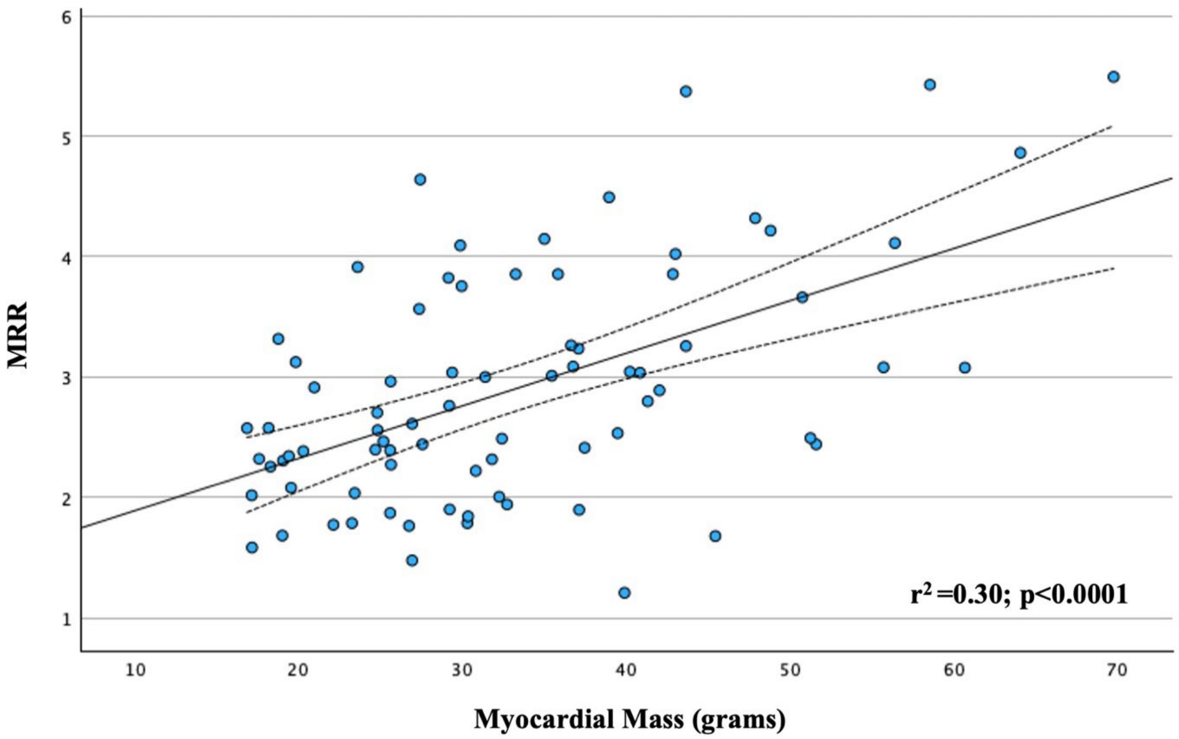

Supplementary Figure 1. Scatterplot of myocardial mass and microvascular resistance reserve (MRR). The solid black line represents the line of best fit and the dashed black lines represent the 95% CI for the line of best fit ($r^2 = 0.30$; $p < 0.0001$).

S3. Pressure derived indices by stenosis location in Isolated LMCA Disease cohort

Coronary luminal imaging was not performed to definitively exclude the presence of occult CAD. Consequently, in the Isolated LMCA Disease cohort, subtle extensions of LMCA disease into the LAD or LCx vessels may not have been fully identified, despite a comprehensive pullback pressure gradient assessment. Hence, a sensitivity analysis was performed discern if the location of LMCA disease impacted coronary physiological measurements. This demonstrated consistent results across varying locations of isolated LMCA disease (ostial, mid-shaft, and distal LMCA) - **Supplementary Table 2.**

Supplementary Table 2. Pressure derived indices by stenosis location in Isolated LMCA Disease cohort

	**LAD FFR**	**LCx FFR**	**P-value**	**LAD iFR**	**LCx iFR**	**P-value**
**Ostial LMCA**	0.77 ± 0.09	0.82 ± 0.09	<0.001	0.88 ± 0.08	0.93 ± 0.07	<0.001
**Mid-LMCA**	0.65 ± 0.12	0.74 ± 0.12	<0.01	0.79 ± 0.12	0.86 ± 0.10	0.02
**Distal LMCA**	0.75 ± 0.12	0.82 ±0.11	<0.001	0.82 ± 0.16	0.89 ± 0.15	<0.001
